# A Novel Role of IGFBP7 in Mouse Uterus: Regulating Uterine Receptivity through Th1/Th2 Lymphocyte Balance and Decidualization

**DOI:** 10.1371/journal.pone.0045224

**Published:** 2012-09-17

**Authors:** Zhen-Kun Liu, Rong-Chun Wang, Bing-Chen Han, Ying Yang, Jing-Pian Peng

**Affiliations:** 1 State Key Laboratory of Reproductive Biology, Institute of Zoology, Chinese Academy of Sciences, Beijing, China; 2 Graduate University of the Chinese Academy of Sciences, Beijing, China; Otto-von-Guericke University Magdeburg, Germany

## Abstract

Previously we have screened out Insulin-like Growth Factor Binding Protein 7 (IGFBP7) as a differentially expressed gene in post-implantation uterus versus pre-implantation uterus by suppressive subtractive hybridation. However its function in uterus was not clearly identified. In this research, the expression and function of IGFBP7 during post-implantation were studied. We found that IGFBP7 was mainly located in the glandular epithelium and the stroma, and was upregulated after embryo implantation. The vector pCR3.1-IGFBP7-t expressing partial IGFBP7 was constructed. Inhibition of IGFBP7 by specific DNA immunization induced significant reduction of implanted embryos and pregnancy rate. The number of implanted embryos (5.68±0.46) was significantly reduced after immunization with pCR3.1-IGFBP7-t, as compared with that of the mice immunized with the control vector (12.29±0.36) or saline (14.58±0.40) (p<0.01). After specific inhibition of IGFBP7, the T helper type 1 (Th1) cytokine IFNγ, was significantly elevated (p<0.05) and the Th2 cytokines IL-4 and IL-10, were reduced in uteri (p<0.05). The increase of Tbet and the decrease of Gata3 were found in mice peripheral lymphocytes by flow cytometry. The expression of decidualization marker IGFBP1 and angiogenesis regulator VEGF were declined in uteri (p<0.05). The expression of apoptosis-associated proteins, caspase3 and Bcl-2, were also declined (p<0.05). These results showed that inhibition of IGFBP7 induced pregnancy failure by shifting uterine cytokines to Th1 type dominance and repressing uterine decidualization.

## Introduction

IGFBP7 is a 31-kD secretory protein that is also known as IGFBP-related protein 1 (IGFBP-rP1), mac25, TAF or angiomodulin [1]. IGFBP7 has an N-terminal IGFBP domain, which shares a high homology with other IGFBPs (IGFBP 1–6) but exhibits a low affinity for IGF [2], and an immunoglobulin domain at the C-terminus [3]. IGFBP7 is widely known as a tumor suppressor gene and is mostly downregulated in many types of cancers [4], [5], [6], [7]. IGFBP7 mRNA is found in uterine glandular epithelial cells and endometrial stromal cells (ESCs), and the mRNA expression is elevated from the mid to late secretory phase of the menstrual cycle in women [8]. In vitro studies have revealed that IGFBP7 functions as a decidualization modulator in endometrial stromal cells [9], [10]. In human umbilical vein endothelial cells, IGFBP7 treatment suppressed extrinsic VEGF-induced tube formation, proliferation, and the phosphorylation of mitogen-activated protein kinase kinase (MEK) and extracellular signal-regulated kinase (ERK) 1/2 [11].

Embryo implantation, which is an important restricting factor for a successful pregnancy, is a cross-talking process that consists of trophoblast invasion into the maternal endometrium and the formation of maternal uterine receptivity [12]. Approximately 75% of pregnancy failures are due to abnormal embryo implantation and placenta formation [13]. Our previous research has elucidated that IGFBP7 regulates the human trophoblast proliferation and invasion [14]. Multiple biological processes are of key importance for embryo implantation. Decidual cells are differentiated from ESCs with the secretion of IGFBP1 [15], and they perform functions in producing growth factors and cytokines, regulating maternal immune responses and restricting trophoblast invasion [12]. Furthermore, embryo implantation relies on extensive vascular remodeling in the endometrial stroma to provide enough nutrients and oxygen for the growing embryos [16], and uterine decidualization and angiogenesis are crucial for the establishment and maintenance of uterine receptivity [17], [18].

In this study, the expression and function of IGFBP7 in uterus was studied by a specific DNA immunization containing truncated IGFBP7 (IGFBP7-t) cDNA in mice. The post-implantation pregnancy failure was significantly higher in the mice immunized with IGFBP7-t expressing plasmid, pCR3.1-IGFBP7-t. We also found that a shift of the cytokine balance to Th1 type dominance and defective stromal decidualization were involved in the pregnancy failure induced by inhibition of IGFBP7.

## Materials and Methods

### Animals

Eight-week-old, sexually mature Kunming mice were purchased from the Experimental Animal Center of the Chinese Academy of Medical Sciences. The mice were housed under conditions of controlled temperature with a 12-h light/dark cycle and free access to water and food. All of the procedures of the animal experiments were approved by the Institutional Animals Care and Use Committee of the Institute of Zoology, Chinese Academy of Sciences.

### Antisera Acquisition and Fertility Efficacy Assay

The healthy and sexually mature female mice were randomly divided into three groups. All of the mice were injected with 100 µl of 0.25% bupivacaine-HCl by multi-spot injections in the leg quadriceps muscle. After 24 h, the mice of the treatment group were injected with 50 ng of pCR3.1-IGFBP7-t (the construction and validation procedure of pCR3.1-IGFBP7-t were shown in Fig. S1) in 100 µl saline (n = 60), and the mice of the control groups were injected with 50 ng of pCR3.1 in 100 of µl saline (control 1, n = 45) or with 100 µl saline (control 2, n = 45). The immunizations were performed three times, with one-week intervals. For each group, blood and muscle samples of five mice were collected one week after the last immunization.

After the last immunization, the female mice were caged with males at a ratio of 2∶1. Day 1 of pregnancy (D1) was validated by the presence of a vaginal plug the next morning. The uteri of pregnant mice sacrificed at D6 or D7 were excised and frozen in liquid nitrogen for further analysis. The numbers of embryos were calculated, photographed and compared with those of the control groups.

### Enzyme-linked Immunosorbent Assay

To evaluate the titer of antibody against IGFBP7 in antisera, a 96-well micro-titer plate (20 ng/well) was coated with recombinant IGFBP7 protein (Peprotech, Rocky Hill, NJ) at 4°C overnight. After blocking with 5% skim milk for 2 h, a serial dilution of the antisera from the mice immunized with pCR3.1-IGFBP7-t, pCR3.1 or saline were incubated with antigen at 37°C for 2 h, followed by incubation with goat anti-mouse horseradish peroxidase-labeled antibody (dilution 1∶10000) at 37°C for 1 h. The plate was then incubated with a Substrate Reaction Pack (R&D, Minneapolis, MN) according to the manufacturer’s instructions, and the optical density (OD) at 450 nm was measured using a Bio-Rad 3550 micro-plate reader (Bio-Rad, Hercules, CA).

Mouse IGFBP1 ELISA kit was used to evaluate the concentration of IGFBP1 in the uteri after immunizations. The protein of mouse uteri after immunizations was incubated with HRP-conjugate reagent in a 96-well micro-titer plate coated with mouse IGFBP1 antibody at 37°C for 60 min. After five times plate washing, the chromogen solution A and B were incubated at 37°C for 15 min. The OD at 450 nm was measured using a Bio-Rad 3550 micro-plate reader (Bio-Rad, Hercules, CA) after adding the stop solution and within 15 min. The concentration was calculated according to the standard concentration and corresponding OD values linear regression equation.

### RNA Extraction and Real-time PCR

The total RNA of the tissue samples was extracted using the Trizol reagent (Invitrogen, CA).

The cDNA synthesis was performed with Moloney murine leukemia virus reverse transcriptase (MMLV) (Promega Corporation, Madison, WI) according to the manufacturer’s instructions using a PTC-100™ programmable thermal controller (MJ Research Inc., MA).

A two-step real-time PCR reaction was performed using a Rotor-gene Q (Qiagen, Hilden) with SYBR Green PCR master mix reagents (Takara Biotechnology, Dalian). After pre-denaturation at 95°C for 10 s, the forty-cycle reaction included denaturation at 95°C for 10 s and extension at 60°C for 60 s. The quantification of IGFBP7 expression was analyzed by the ΔΔC_t_ method. The primers used for IGFBP7 and GAPDH were as follows: qIGFBP7 forward, AAGAGGCGGAAGGGTAAAGC; qIGFBP7 reverse, TGGGGTAGGTGATGCCGTT; qGAPDH forward, CATGAGAAGTATGACAACAGCCT; and qGAPDH reverse, AGTCCTTCCACGATACCAAAGT.

### Immunofluorescence

Hela cells were cultured in Dulbecco’s Modified Eagle Medium (Invitrogen, CA), seeded on microscope slides for 24 h, and transfected with the pCR3.1-IGFBP7-t plasmid using lipofectamine 2000 (Invitrogen, CA). The Hela cells were then fixed in 4% paraformaldehyde and permeabilized in a 0.1% sodium citrate/0.1% Triton X-100 solution at 48 h after the transfection. The transfected and non-transfected Hela cells were blocked in 5% bovine serum albumin (BSA) (Invitrogen, CA) and then incubated with mouse sera at 4°C overnight. The cells were then incubated with rabbit anti-mouse IgG conjugated with FITC (Jackson, PA) at 37°C for 1 h and counterstained with prodium iodide (PI) (Sigma, MO). The slides were observed and photographed using an LSM 510 META (Zeiss, NY) microscope.

### Immunohistochemistry (IHC)

The general procedures for the IHC were as follows: frozen sections of mouse uteri were collected on 3-aminopropyltriethoxy-silane-coated slides in 4% paraformaldehyde for 30 min. After treatment with 3% H_2_0_2_ for 10 min, the sections were blocked with 5% BSA at 37°C for 1 h and then incubated with rabbit anti-IGFBP7 (sc-13095, Santa Cruz, CA) and rabbit anti-VEGF antibody (Millipore, MA) at 4°C overnight. The sections were then incubated with goat anti-rabbit IgG conjugated HRP (KPL, Gaithersburg, MD) (dilution 1∶200) at 37°C for 30 min. The color reaction was developed with the addition of diaminobenzidine tetrahydrochloride (Sigma-Aldrich, MO). The sections were counterstained with hematoxylin (Sigma-Aldrich, MO).

### Western Blotting

A total of 70 µg of denatured protein from the uteri samples was electrophoresed on a polyacrylamide gel and electroblotted onto a nitrocellulose membrane. After blocking with 5% non-fat milk for 1 h, the membranes were incubated with the following primary antibodies at 4°C overnight: rabbit anti-IGFBP7 (sc-13095, Santa Cruz, CA), rabbit anti-IGFBP1 (sc-13097, Santa Cruz, CA), rabbit anti-VEGF (Cat. # 07–1420, Millipore, MA), rabbit anti-TGFβ (AB64715-100, Abcam, MA), goat anti-IL-4 (sc-1260, Santa Cruz, CA), goat anti-IL-10 (sc-1783, Santa Cruz, CA), rabbit anti-IFNγ (15365-1-AP, Proteintech, IL), mouse anti-Bcl-2 (sc-7382, Santa Cruz, CA), mouse anti-Bax (sc-7480, Santa Cruz, CA), rabbit anti-FAS (sc-715, Santa Cruz, CA), rabbit anti-FAS-L (sc-834, Santa Cruz, CA), rabbit anti-caspase3 (sc-7148, Santa Cruz, CA), or mouse anti-β-Actin (sc-47778, Santa Cruz, CA). The membranes were then incubated with the following secondary antibodies (dilution 1∶5000) at 37°C for 1 h: Dylight™ 800-labeled goat anti-mouse, Dylight™ 700-labeled goat anti-mouse, Dylight™ 800-labeled goat anti-rabbit, or Dylight™ 800-labeled donkey anti-goat (KPL, Gaithersburg, MD). The relative protein quantification was analyzed using the Odyssey infrared imaging system v3.0 (LI-COR, Lincoln, NE) according the manufacturer’s instructions.

### Flow Cytometry

On D7 of pregnancy, immunized mice were sacrificed. The peripheral blood lymphocytes were isolated by Histopaque (Sigma-Aldrich, St. Louis, MO). Cells were stained with anti-human/mouse Tbet PE antibody (Ebioscience, San Diego, CA) and anti-human/mouse Gata3 Alexa fluor 647 antibody (Ebioscience, San Diego, CA). Data were acquired with a FACSvantage Diva (BD, Franklin Lakes, NJ) within 24h staining and further analyzed by the Flowjo 6.4.1 software (Tree Star, Ashland, OR).

### Statistics

Statistical analysis was performed using one-way analysis of variance (ANOVA) or an independent-samples t-test. All of the values are displayed as the mean ± SEM. P<0.05 indicated a significant difference and P<0.01 indicated a highly significant difference.

## Results

### Expression of IGFBP7 in Post-implantation Mouse Uteri

**Figure 1:**
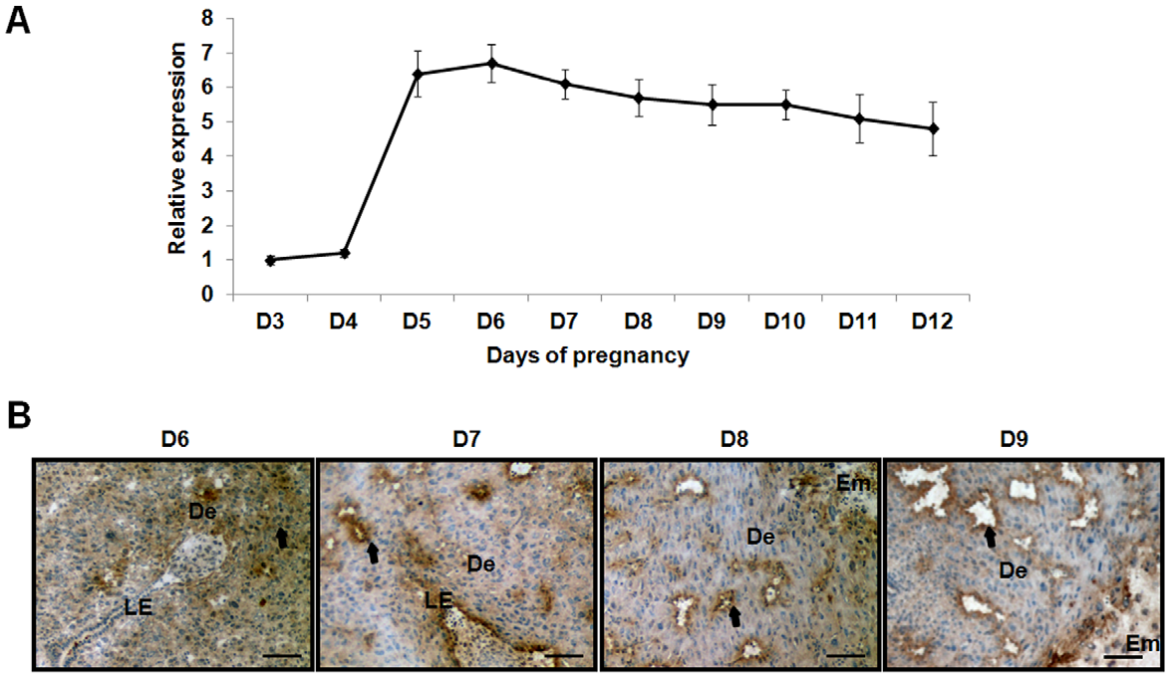
The expression of IGFBP7 during the post-implantation period. A: The expression of IGFBP7 in uteri from D3 to D12 of pregnancy. The vertical axis represents the relative expression of IGFBP7 normalized to internal control GAPDH. The expression of IGFBP7 was found to be dramatically elevated and was maintained at a relative high level after D5 of pregnancy. B: In situ expression of IGFBP7 in uteri from D6 to D9 of pregnancy. The brown staining represents positive signals for IGFBP7. IGFBP7 was expressed in the glandular epithelium and stroma. Bar represents 250 µm. LE: Luminal epithelium; De: Decidua; Em: Embryo.

The real-time PCR analysis showed that the expression of IGFBP7 was dramatically elevated and maintained at a high level at D5 of the pregnancy and thereafter (Fig. 1A). The IHC results showed that IGFBP7 was mainly located in the glandular epithelium and stroma from D6 to D9 pregnancy (Fig. 1B).

### Stimulation of the Anti-IGFBP7 Antibody in Mice after Immunization with pCR3.1-IGFBP7-t

**Figure 2:**
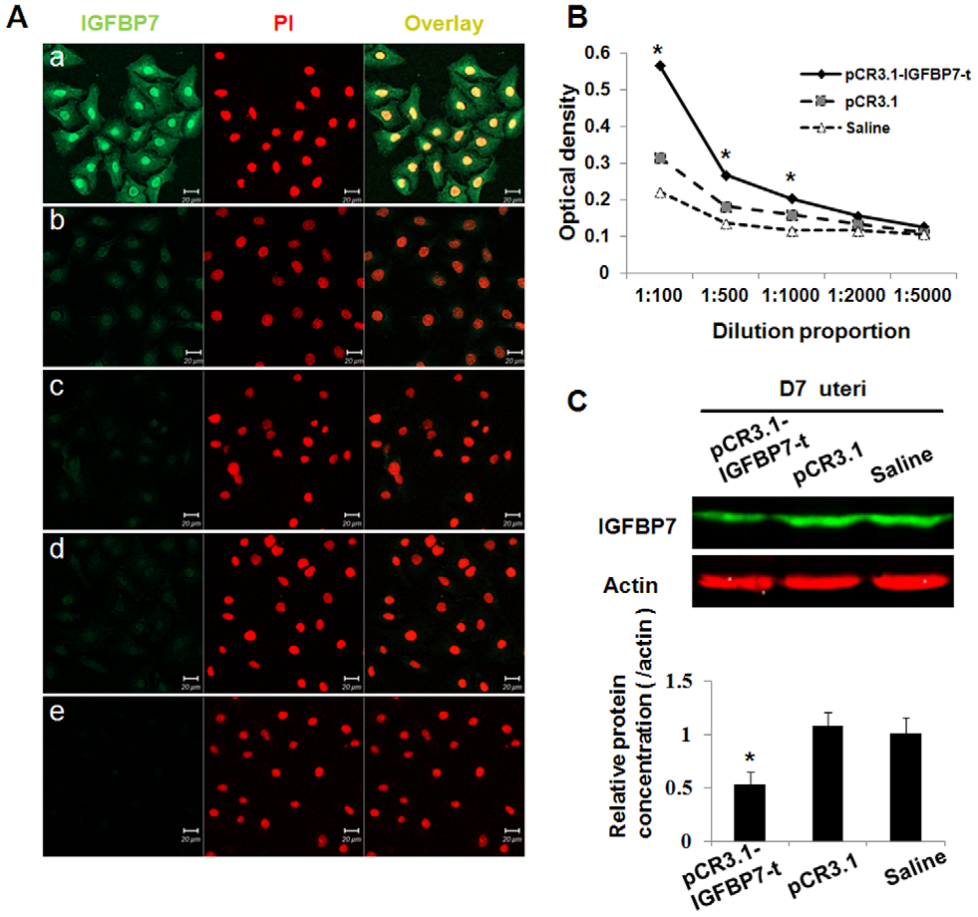
Detection and function of anti-IGFBP7 antibody in mice after immunizations. A: Immunofluorescence detection of pCR3.1-IGFBP7-t expression in transfected Hela cells. Nuclei were stained with PI. a, b, c: Hela cells transfected with the pCR3.1-IGFBP7-t plasmid were incubated with antisera from mice immunized with pCR3.1-IGFBP7-t, pCR3.1 or saline respectively. d, e: Non-transfected Hela cells were incubated with antisera from mice immunized with pCR3.1-IGFBP7-t or saline respectively. B: Detection of anti-IGFBP7 antibody by Elisa assay. Antisera from mice immunized with pCR3.1-IGFBP7-t, pCR3.1 or saline were serially diluted from 1∶100 to 1∶5000. C: Western blotting analysis of IGFBP7 expression in the uteri of pregnant mice at D7. Actin served as an internal control. The relative expression of IGFBP7/actin in the uteri of the mice immunized with pCR3.1-IGFBP7-t, the pCR3.1 vector or saline were shown. IGFBP7 was significantly reduced after immunization with pCR3.1-IGFBP7-t. Bar represents 100 µm. *: P<0.05.

The construction and validation of the pCR3.1-IGFBP7-t plasmid were shown in Fig. S1. To obtain antisera, the pCR3.1-IGFBP7-t and control vectors were injected into the muscles of the mice. The expression of the IGFBP7-t recombinant protein was confirmed by the specific reaction of the antisera from the mice immunized with pCR3.1-IGFBP7-t and the Hela cells transfected with pCR3.1-IGFBP7-t (Fig. 2A a). No positive signal was detected for the Hela cells transfected with pCR3.1-IGFBP7-t when the antisera from the mice immunized with pCR3.1 or saline were used (Fig. 2A b and c). These results showed that an anti-IGFBP7 antibody was generated by the immunization with pCR3.1-IGFBP7-t. Panel c and d in Fig. 2A show that the antisera from pCR3.1-IGFBP7-t- or saline-treated mice did not react with non-transfected Hela cells.

After three immunizations, the titer of the sera from the mice immunized with pCR3.1-IGFBP7-t was higher than 1∶1000 by ELISA (Fig. 2B). The expression of the IGFBP7 protein was notably attenuated after treatment in the uteri compared with the controls (Fig. 2C). These results indicated that the anti-IGFBP7 antibodies were successfully produced after the immunization with pCR3.1-IGFBP7-t. And the expression of IGFBP7 in uterus was significantly reduced by specific DNA immunization.

### Pregnancy Failure during Post-implantation Induced by the Inhibition of IGFBP7 in Mice

**Figure 3:**
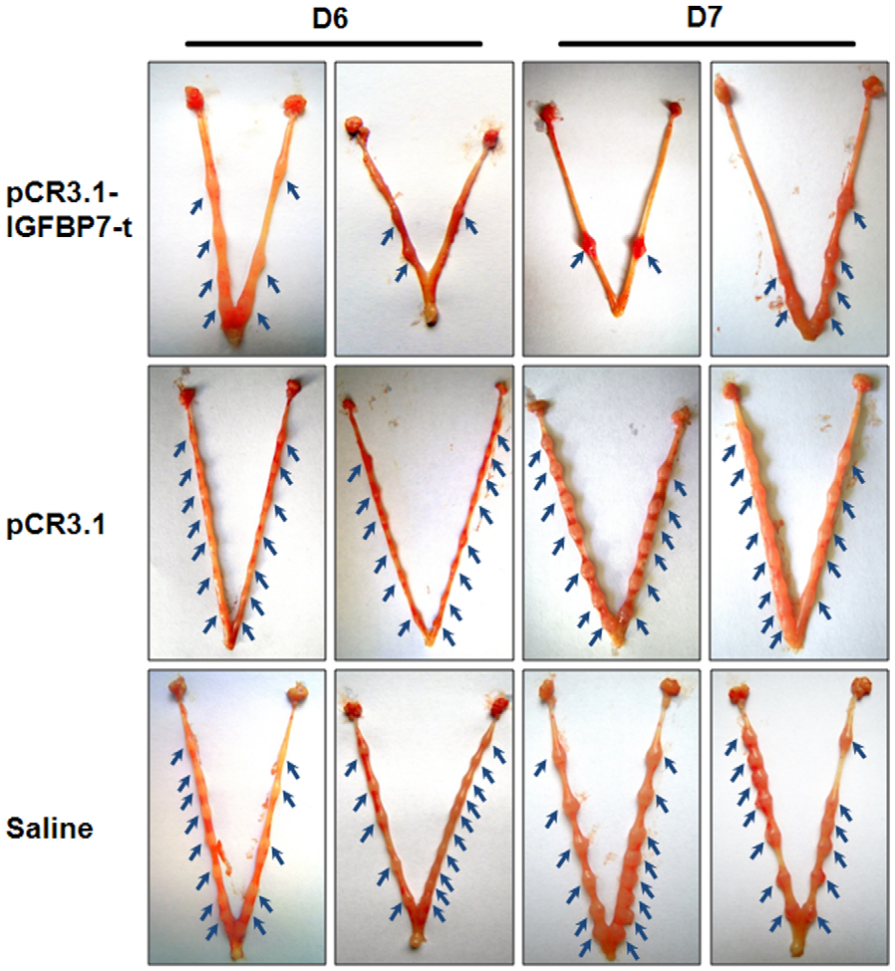
The implanted embryos of mice after immunizations. Uteri from pregnant mice (day 6 and day 7) were removed and the implanted embryos were counted. The arrows indicated implanted embryos. A significant reduction of embryos was observed in mice immunized with pCR3.1-IGFBP7-t.

After treatment, the female mice showed partial pregnancy failure throughout the entire observation period. The pregnancy rate of the mice receiving pCR3.1-IGFBP7-t immunization was reduced to 60% of the control levels (Table 1). The number of implanted embryos (5.68±0.46), as indicated by blue arrows in Fig. 3, was significantly reduced (p<0.01) on D6 and D7 after immunization with pCR3.1-IGFBP7-t, as compared with that of the mice immunized with the control vector (12.29±0.36) or saline (14.58±0.40) (Table 1).

**Table 1:** Statistics of pregnancy rate and implanted embryos.

Groups	N	Rp	Nf
pCR3.1-IGFBP7-t	60	60%	5.68±0.46**
pCR3.1	45	91.1%	12.29±0.36
Saline	45	100%	14.58±0.40

N: the number of mice receiving immunization in this experiment. Rp: the pregnancy rate, the ratio of the number of pregnant mice to N. Nf: the average number of embryos in the uterus of one mouse.

** P<0.01.

### Effect of IGFBP7 on Peripheral Lymphocyte Balance and Th-associated Cytokines Expression in the Uterus

**Figure 4:**
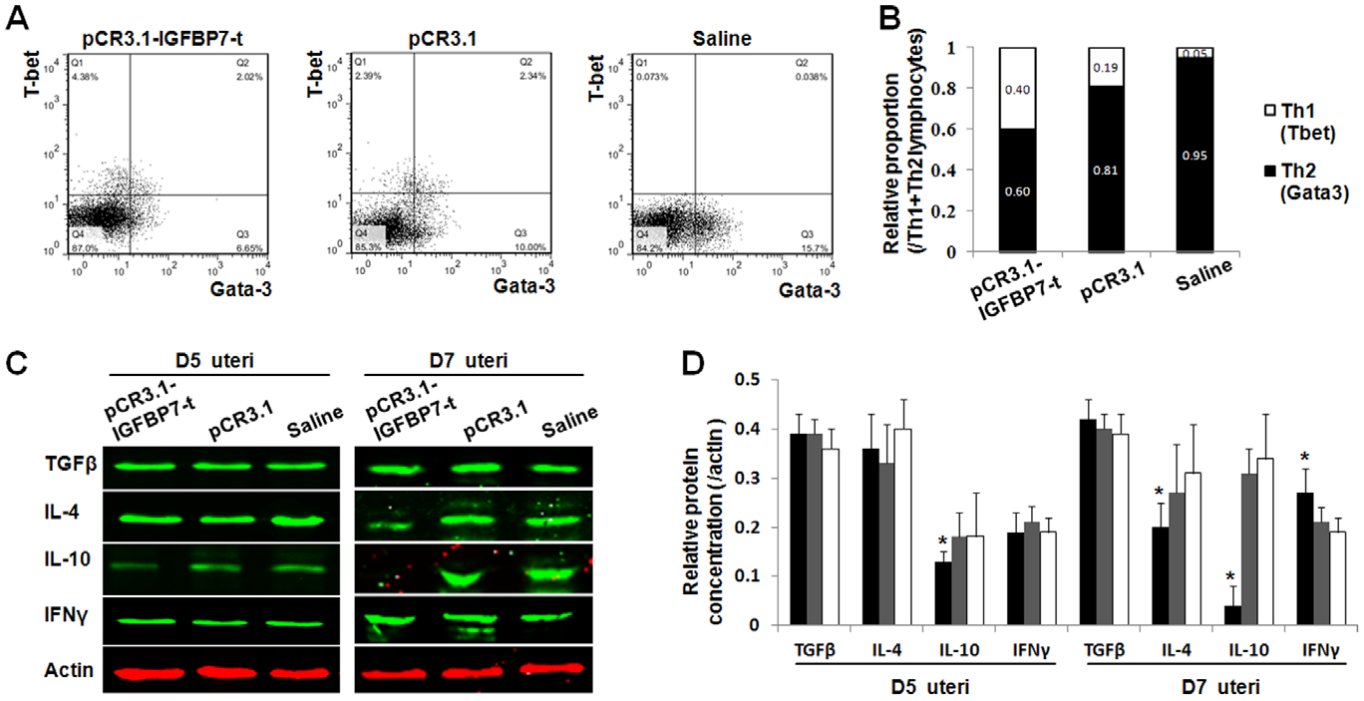
The analysis of uterine cytokines and T helper cells differentiation markers in mice peripheral lymphocytes after immunizations. A: The flow cytometry detection of Tbet and Gata3 expression. The expression of Tbet was increased while the expression of Gata3 was decreased in the mice after immunization with pCR3.1-IGFBP7-t. B: the ratio of differentiated Th1 and Th2. Th1 was represented by Tbet, while Th2 was represented by Gata3. The relative proportion of Tbet (Q1) and Gata3 (Q3) were counted and shown. C: Western blotting analysis of uterine cytokines. After immunization with pCR3.1-IGFBP7-t, the expression of Th1 type cytokine IFNγ was significantly elevated and Th2 type cytokines IL-4, IL-10 were declined. No significant variation of TGFβ was observed. D: The statistics of relative expression of cytokines to actin. The black columns represent the relative expression value in mice immunized with pCR3.1-IGFBP7-t, whereas the grey columns represent the mice immunized with pCR3.1, and the white columns indicate the mice immunized with saline. *: P<0.05.

In order to identify the molecules that are involved with pregnancy failure, the key cytokines that regulate uterus receptivity were examined in the uteri of immunized mice at D5 and D7 of pregnancy (Fig. 4C and D). At D7 of pregnancy we found that the expression of cytokines was altered: Th2 cytokines IL-4 and IL-10 were decreased; Th1 cytokine IFNγ was elevated in the mice immunized with pCR3.1-IGFBP7-t. There was no notable change in the TGFβ levels among the different groups. At D5 of pregnancy, the expression of IL-10 was decreased, while other molecules were not notably altered (Fig. 4D). These results suggest an alternation of immune balance after inhibition of IGFBP7. To confirm our results, the expression of a marker of Th1 differentiation Tbet, and a marker of Th2 differentiation Gata3 at D7 of pregnancy were detected by flow cytometry (Fig. 4A). The increase of Tbet and the decrease of Gata3 (Fig. 4B) suggest the ratio of Th1/Th2 lymphocytes was biased to Th1 dominance after immunization with pCR3.1-IGFBP7-t, which is consistent with the western blotting results.

### Effect of IGFBP7 on Decidualization-associated Molecules in the Uterus

**Figure 5:**
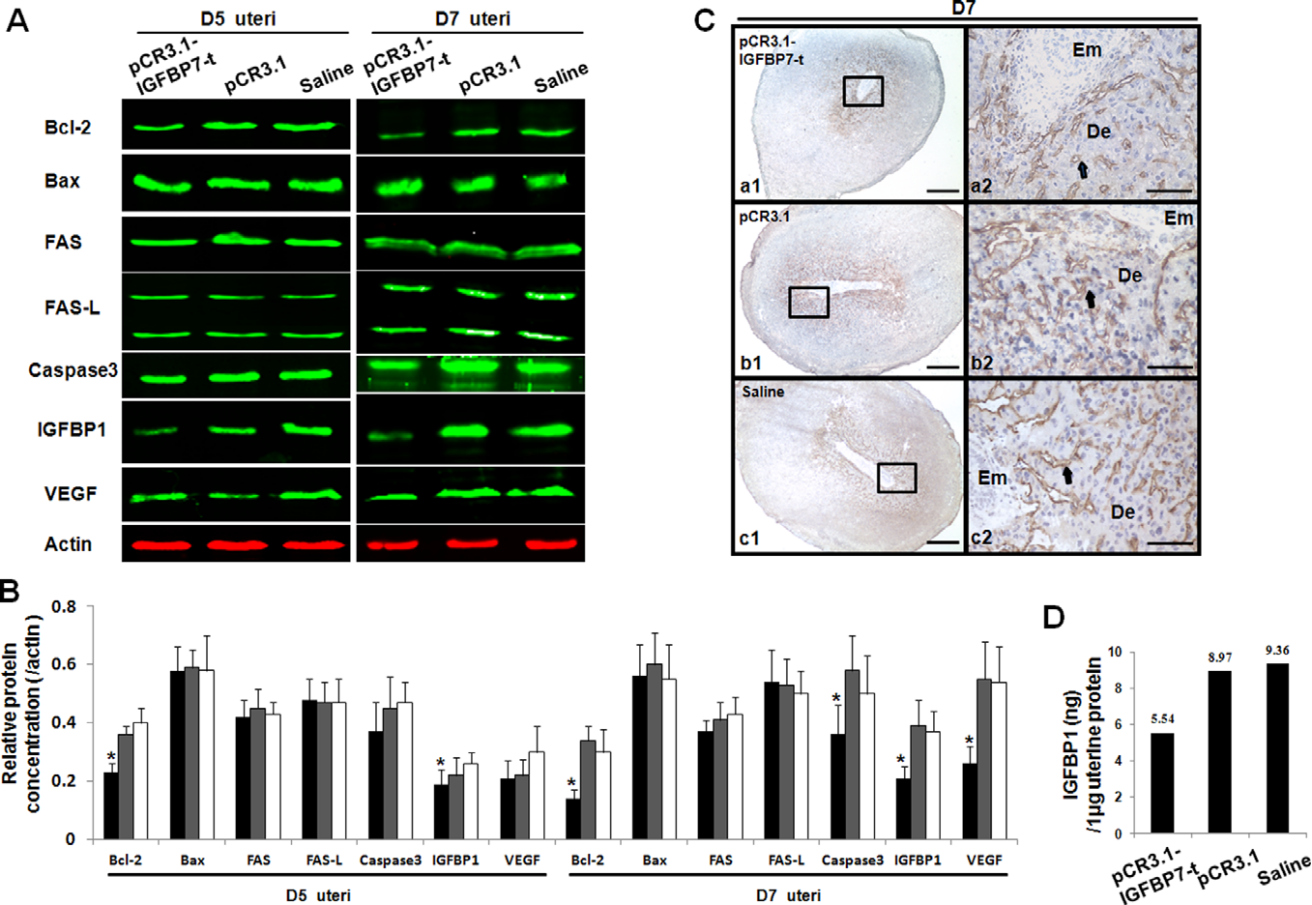
The analysis of uterine decidualization-associated molecules. A and B: the relative expression of target proteins in the D7 uteri of mice immunized with pCR3.1-IGFBP7-t, the pCR3.1 vector or saline. The black columns represent the relative expression value in mice immunized with pCR3.1-IGFBP7-t, whereas the grey columns represent the mice immunized with pCR3.1, and the white columns indicate the mice immunized with saline. After immunization with pCR3.1-IGFBP7-t, the expression of Bcl-2, caspase3, IGFBP1 and VEGF were significantly decreased. No significant variation of Bax, FAS or FAS-L was observed. C: Reduced expression of VEGF in mice uteri at D7 after immunization with pCR3.1-IGFBP7-t. Brown staining represents the positive signals for VEGF, which was found to be reduced after DNA immunization. De: Decidua; Em Embryo. Bar represents 250 µm in a1, b1 and c1 and 50 µm in a2, b2 and c2. D: The quantitation of IGFBP1 in uterus by ELISA. After immunization with pCR3.1-IGFBP7-t, the expression of IGFBP1 in uterus was decreased to about 5.54 ng/1 µg uterine protein. *: P<0.05.

After immunization with pCR3.1-IGFBP7-t, the apoptosis-associated molecules Bcl-2 and caspase3 in uteri were decreased, whereas Bax, FAS and FAS-L were not altered relative to the controls at D7. The decidualization marker IGFBP1, and the key regulator of angiogenesis VEGF, were significantly depleted in the mice immunized with pCR3.1-IGFBP7-t at D7. At D5 Bcl-2 and IGFBP1 were decreased, while the others were not altered (Fig. 5A and B). According to the IHC experiment, VEGF was located in the vascular endothelial cells and was depleted in the mice immunized with pCR3.1-IGFBP7-t (Fig. 5C). The concentration of IGFBP1 in mice immunized with pCR3.1-IGFBP7-t was dramatically decreased to 5.54 ng/1 µg uterine protein (Fig. 5D).

## Discussion

Successful pregnancy in primates requires a well-established decidua and an adaptable immune microenvironment. In the mouse, embryos implant into the uterus at D4.5 in a normal pregnancy [12]. A series of cell transformation and differentiation events then takes place, and immediately after implantation, ESCs transform into decidual cells to establish uterine receptivity. In vitro studies have shown that IGFBP7 is expressed and secreted in ESCs, and the inhibition of IGFBP7 in ESCs induces the decrease of IGFBP1 and prolactin [9], [10]. However, the role of IGFBP7 in vivo has yet to be clearly elucidated. In this study, we constructed a vector, pCR3.1-IGFBP7-t, containing a truncated IGFBP7 coding sequence in order to investigate the effect of IGFBP7 on uterine receptivity and pregnancy in female mice. The immunizations were based on our previous reports [19], [20] and involved pre-inoculation with the muscle-damaging agent bupivacaine at the injection sites one day before the DNA immunization. Our in vitro validation of the expression and specificity showed the successful expression of pCR3.1-IGFBP7-t mRNA, and the antibody generated by the immunization with pCR3.1-IGFBP7-t specifically reacted with the IGFBP7-t expressed by pCR3.1-IGFBP7-t in transfected Hela cells. Both the pregnancy rate and number of implanted embryos were significantly reduced after the immunization with pCR3.1-IGFBP7-t.

The immune microenvironment of the uterus is crucial for the maintenance of pregnancy, and cytokines are considered to be key regulators [21]. Previously pregnancy was recognized as a predominant Th2 immunity event, which may protect the fetus from being attacked by Th1 cells [22]. However, predominant Th2 type immunity was found in abortion cases [23], and the typical Th2 type cytokines IL-4 and IL-10 KO mice are fertile [24]. These evidences indicate that Th2 dominance is insufficient in pregnancy [25]. Now the Th1/Th2 paradigm has been expanded into Th1/Th2/Th17 and regulatory T (Treg) cells paradigm [26]. Th17 cells play a role in inflammation [27], while Treg cells are important in immunoregulation and immunotolerance [28], [29]. The differentiation of both Th17 and Treg cells from naive T cells require the involvement of TGFβ. In our data there is no notable change of TGFβ among different groups, which may indicate that the differentiation of Th17 and Treg cells were not significantly influenced by inhibition of IGFBP7. The increase of Th1 differentiation marker Tbet and the decrease of Th2 differentiation marker Gata3 implicated that the Th1/Th2 ratio is biased to Th1 dominance after immunization of pCR3.1-IGFBP7-t, and also confirmed the expression profile of the Th1 and Th2 cytokines. At D7 we observed that IFNγ was elevated, whereas Th2 cytokines IL-4 and IL-10 were decreased in the uterus after the immunization with pCR3.1-IGFBP7-t. IL-10 is not only a typical Th2 cytokine, but also plays an important role in Treg functioning [29]. Moreover, IL-10 also regulates the vascular remodeling and hypertension to maintain pregnancy [30]. IL-10 could rescue intrauterine growth restriction and proteinuria in IL-10^−/−^ mice [31]. The uNK-derived IFNγ facilitate the gestation spiral aterial modification [32]. However, excessive IFNγ is deleterious to pregnancy. Therefore, the expressions of both the transcription factors and cytokines exhibited a tendency toward the diminution and exclusion of the fetus.

IGFBP1 is recognized as a decidualization marker [10]. In our study, IGFBP1 was significantly reduced in mice at D7 after immunization with pCR3.1-IGFBP7-t, as compared with the controls. This result implicates the insufficient formation of decidual cells as a possible factor that contributes to the partial pregnancy failure. However, apoptosis is involved in the establishment of uterine receptivity, and specific uterine cells undergo apoptosis during decidualization [33]. Caspase3, the final effector protein of apoptosis, was downregulated at D7 after immunization with pCR3.1-IGFBP7-t. Bcl-2, which is well known as an apoptosis-inhibiting protein, was also downregulated at D7. The ratio of Bax/Bcl-2 partially contributes to the cell fate in the mitochondrial apoptosis pathway [34]; however, Bcl-2 also functions as a cell cycle inhibitor [35]. These results implicated an attenuated apoptosis process in the uteri after the immunization with IGFBP7, which may also contribute to insufficient decidualization. Furthermore, a notable decrease of VEGF was also observed in the pregnant mice immunized with pCR3.1-IGFBP7-t at D7. VEGF is essential for angiogenesis through the regulation of endothelial proliferation and migration [36]; hence, a high expression of VEGF is beneficial for the growth of the embryos and placenta [37], [38], whereas a low expression may contribute to inadequate embryo implantation or development.

According to our previous research, the effect of DNA immunization was released gradually after immunizations [39]. As the immunizations were prior to the mating of mice, the cumulative effect after immunization with pCR3.1-IGFBP7-t may impair the implantation process. We found out that three molecules, IL-10, Bcl-2 and IGFBP1, were downregulated on D5, while the upregulation of IFNγ and the downregulation of IL-4, Caspase-3 and VEGF were also observed on D7. This gradual impact on the molecules could partially stand for it. However the further mechanism of IGFBP7 on implantation is still worth devoting in. Overall, we observed alterations in the uterine environment after the immunization with pCR3.1-IGFBP7-t, which was generally disadvantageous for pregnancy maintenance. As the establishment of uterine receptivity is a complex process, it remains unclear whether the observed physiological and immunological changes are directly due to the interference of IGFBP7 or are secondary effects on decidualization induced by the loss of IGFBP7. The inhibitory effects of pCR3.1-IGFBP7-t immunization on pregnancy genes may not be restricted to the uterus despite the fact that we removed the sequence coding for the highly conserved IGFBP domain in the construction of pCR3.1-IGFBP7-t to minimize any off-target effects. Similarly, the impact of the suppression of IGFBP7 on the neonates requires further study. In conclusion, we found that IGFBP7 was mainly expressed in the glandular epithelium and stoma and was significantly upregulated during the post-implantation period. Immunization with pCR3.1-IGFBP7-t could reduce the embryo implantation and pregnancy rates in mice by generating anti-IGFBP7 antibodies. After immunization with pCR3.1-IGFBP7-t, the decidualization in pregnant uterus was repressed, and the balances of cytokines and lymphocytes were shifted to Th1 dominance.

## Supporting Information

Figure S1 **Construction and validation of the plasmid pCR3.1-IGFBP7-t.** A: Schematic diagram of pCR3.1-IGFBP7-t. A partial sequence of IGFBP7 (NM_008048.3, CDS: 253 bp-849 bp) containing the functional domains was cloned from mouse uterus using primers with HindIII/KpnI restriction sites (the forward primer with the HindIII digestion site, CCCAAGCTTGATGGAGTGCGTGAAGAGC; the reverse primer with the KpnI digestion site, CGGGGTACCTTATAACTGAGCACCTTCACC). The sequence was then cloned into the pCR3.1 vector B: Enzyme digestion. Lane 1 shows pCR3.1-IGFBP7-t before HindIII/KpnI digestion. Lane 2 shows pCR3.1-IGFBP7-t digested with HindIII/KpnI for 2 h. The band between 500 bp and 750 bp represents the inserted sequence. C: The mRNA expression of pCR3.1-IGFBP7-t in transfected Hela cells. The cDNA of Hela cells transfected with pCR3.1-IGFBP7-t or pCR3.1 and non-transfected cells were used as the templates for PCR amplification. GAPDH served as the internal control. D: The mRNA expression of pCR3.1-IGFBP7-t in the muscle of immunized mice. To obtain antisera, the pCR3.1-IGFBP7-t and control vectors were injected into the muscles of the mice. After immunization, the IGFBP7 mRNA expression of the mice immunized with pCR3.1-IGFBP7-t was elevated.
(TIF)
