# Supplementary figures and images for: A Novel Role of IGFBP7 in Mouse Uterus: Regulating Uterine Receptivity through Th1/Th2 Lymphocyte Balance and Decidualization
Source: PLoS One. 2012 Sep 17;7(9):e45224. doi: 10.1371/journal.pone.0045224 (PMC3444470; doi:10.1371/journal.pone.0045224)

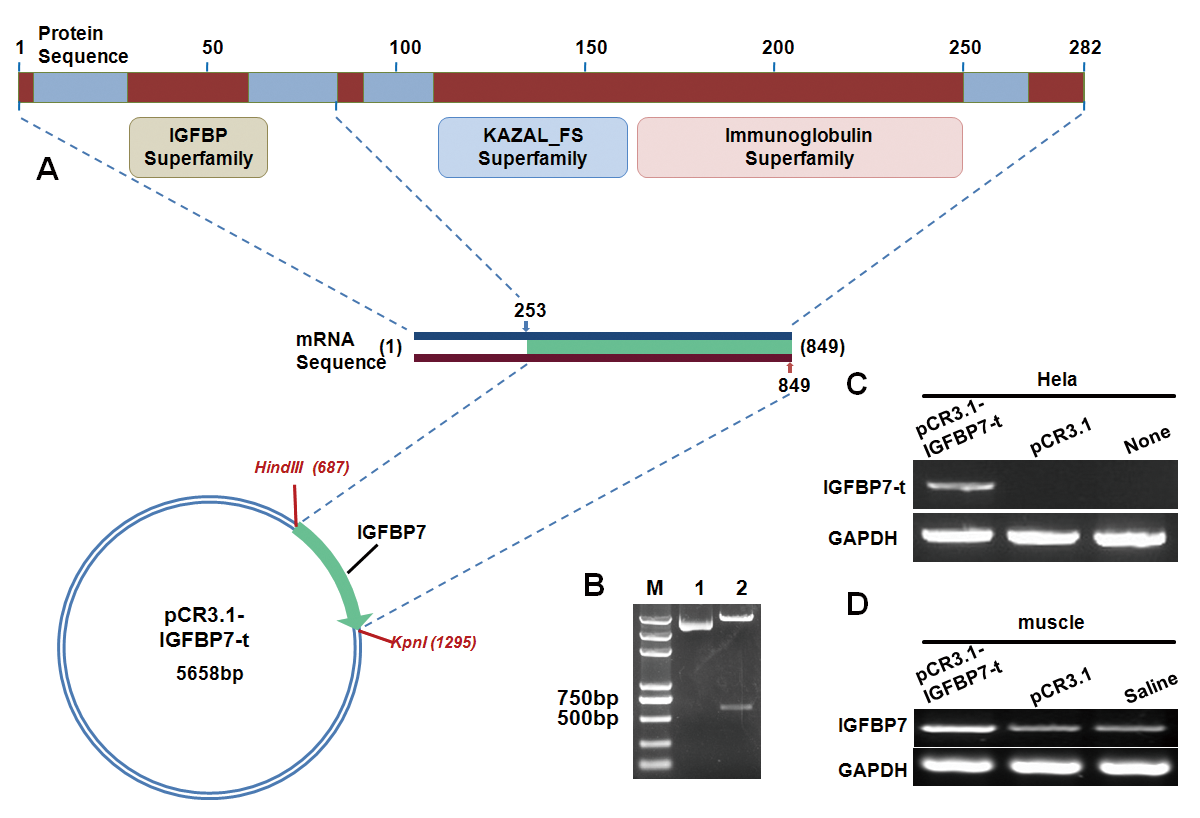

Supplement: Figure S1 — Construction and validation of the plasmid pCR3.1-IGFBP7-t. A: Schematic diagram of pCR3.1-IGFBP7-t. A partial sequence of IGFBP7 (NM_008048.3, CDS: 253 bp-849 bp) containing the functional domains was cloned from mouse uterus using primers with HindIII/KpnI restriction sites (the forward primer with the HindIII digestion site, CCCAAGCTTGATGGAGTGCGTGAAGAGC; the reverse primer with the KpnI digestion site, CGGGGTACCTTATAACTGAGCACCTTCACC). The sequence was then cloned into the pCR3.1 vector B: Enzyme digestion. Lane 1 shows pCR3.1-IGFBP7-t before HindIII/KpnI digestion. Lane 2 shows pCR3.1-IGFBP7-t digested with HindIII/KpnI for 2 h. The band between 500 bp and 750 bp represents the inserted sequence. C: The mRNA expression of pCR3.1-IGFBP7-t in transfected Hela cells. The cDNA of Hela cells transfected with pCR3.1-IGFBP7-t or pCR3.1 and non-transfected cells were used as the templates for PCR amplification. GAPDH served as the internal control. D: The mRNA expression of pCR3.1-IGFBP7-t in the muscle of immunized mice. To obtain antisera, the pCR3.1-IGFBP7-t and control vectors were injected into the muscles of the mice. After immunization, the IGFBP7 mRNA expression of the mice immunized with pCR3.1-IGFBP7-t was elevated. (TIF) [file pone.0045224.s001.tif]
